# Adherence to commercial food thickener in patients with oropharyngeal dysphagia

**DOI:** 10.1186/s12877-023-04589-4

**Published:** 2024-01-16

**Authors:** Amaya Peñalva-Arigita, Maria Lecha, Anna Sansano, Rosa Prats, Aida Vásquez, Helena Bascuñana, Lluis Vila

**Affiliations:** 1 Nutritional & Dietetics Unit, Hospital Moisès Broggi, Oriol, C. d’Oriol Martorell, 12, Sant Joan Despí, Barcelona, 08970 Spain; 2 Nutritional & Dietetics Unit, Hospital Moisès Broggi, C. d’Oriol Martorell 12., Sant Joan Despí, Barcelona, 08970 Spain; 3 Physical Medicine and Rehabilitation Department Sant Pau University Hospital, Sant Quintí, 89, Barcelona, 08041 Spain; 4 Endocrinology and Nutrition Department, Hospital Moisès Broggi, C. d’Oriol Martorell, 12, Sant Joan Despí, Barcelona, 08970 Spain

**Keywords:** Dysphagia, Adherence, Compliance, Persistence, Commercial thickener, Implementation

## Abstract

**Background:**

Oropharyngeal dysphagia (OD), a common symptom in the elderly, uses commercial thickener (CT) as part of its treatment. This is often accompanied of dislike and poor compliance.

**Aim:**

Describe adherence to CT and possible differences according to dwelling location in an area of influence of approximately 400.0000 inhabitants.

**Methods:**

Cohort prospective observational study. Randomized patients from Nutrition and Dietetic (NDU)-database (4 calls-interviews/year). Variables: Age, diagnostic, gender, dwelling/location: Home (H) / Nursing Home (NH), viscosity (nectar, honey, pudding), days with CT. Adherence measured with a questionnaire, considering implementation of treatment by combining CT use and consumption data, categorised in three groups good, moderate and poor. Change in patterns (improvement, maintenance, worsening) and non-adherence reasons.

**Results:**

One hundred sixty-eight patients recruited with indicated viscosity: Nectar 39.7%, honey 29.3% and pudding 30.8%. Average age of 82.6 ± 11.1 years; 57.8% women (46.4% at H vs. 67% at NH, *p* < 0.01). Dwelling/location: 80 (47.6%) live at H and 88 (52.4%) at NH. Days with CT prior study were 509 ± 475.28. Implementation found in first call: good in 50%, moderate in 20.2% and poor in 29.8%. At first call, adherence parameters were more favourable in NH compared to H. However these parameters were reversed during the study period as there was an improvement at H vs. NH. Also in terms of change in patterns a significant improvement of implementation was found in patients living at H, 31.1% vs. those living at NH, 15.7%, *p* < 0.05. CT persistence throughout study was 89.7%.

**Conclusions:**

Low adherence to CT found in our community. Telephone follow-up resulted in improved adherence, especially in the H population. Our data provides valuable insights into the variability and changes in CT adherence among patients with OD. Adherence is complex and subject to many factors and dwelling/location is one of them. This study reveals the need to approach CT treatment for OD differently in NH.

**Supplementary Information:**

The online version contains supplementary material available at 10.1186/s12877-023-04589-4.

## Introduction

 The global human population is ageing. Between 2020 and 2030 the percentage of the world’s population over 60 years of age will increase by 34%, and could double by 2050 [1]. Related to old age may be swallowing disorders such as oropharyngeal dysphagia (OD), one of the lesser-known geriatric syndromes [2]. OD is associated with increased morbidity, mortality and higher hospital costs, affecting around 40% of people over 65 years of age [3]. Furthermore, its incidence increases in patients aged ≥ 80 years [4].

Elderly institutionalization is a global phenomenon and in the last decade the number of geriatric institutions in Spain has increased to 444.000 institutionalised people currently, 79% of whom are > 80 years [5]. Catalonia is one of the communities with the highest number of such institutions and our area of influence (Baix Llobregat with 400,000 inhabitants) represents 10.61% of this [6]. The prevalence of OD found in institutionalised people is between 30% [7] and 70% [8]. At our institution we conducted a study which found a prevalence of 30% in acute hospitalisation and 27% in long term socio-health care [9].

Treatment for OD is complex, requiring a combination of multidisciplinary approaches [10]. As part of the therapeutic strategy, commercial thickener (CT) is used to increase liquid viscosity as it decreases swallowing transit speed [11, 12]. This adaptation of fluids aims to preserve the oral route as much as possible, avoiding dehydration, malnutrition and the risk of aspiration secondary to OD [13]. Its use has been shown to reduce laryngeal vestibule penetrations and tracheo-bronchial aspirations [13–15]. However, there is a related concern with the dislike of thickened water which is possibly accompanied by poor compliance [16]. In relation to this, adherence to CT is a fundamental part of therapeutic intervention, and has been identified as a problem in its management [10]. It has been demonstrated that non-compliance with instructions in OD is followed by adverse effects [17]. In the 1970s Haynes [18] described the terms of a*dherence* and *compliance* which are used to study how well a person follows an indicated health or pharmacological treatment. There is still, no clear terminology around the subject and these terms are interspersed in definitions and in practice are interchangeable [19]. In general, adherence is associated with greater patient involvement together with collaboration from the healthcare provider, and with compliance being associated with more passive patient behaviour linked to health-related obedience [20]. There are yet more terms added in this subject such as persistence, which is defined as the time the patient continues treatment [19], or implementation of treatment defined as the extent to which the actual guideline reported by the patient matches the prescribed one [20]. Particularly in elderly populations factors influencing adherence increase and medication use is not well described despite being a significant cause of morbidity [21]. Overall, there is found to be poor adherence, of 70% in preventive programmes and up to 50% in chronic treatments [22]. Adherence data in OD varies around 22–52% but studies are notably heterogeneous [23, 24].

CT is financed by the Spanish health system, and in Catalonia is dispensed by hospitals wherein each one specifies how it is managed. In ours it is managed jointly by the Nutrition and Dietetic Unit (NDU) and the Pharmacy Service. In day-to-day clinical work we have observed a certain degree of non-compliance for CT among our patients. This prompted us to study adherence to CT treatment in our area of reference. Since the role of CT in preventing complications is clear, and correct use and compliance is desirable, our aim for the study is to find out adherence or compliance in the use of commercial thickener in our population and to identify reasons for not compliance, as well as to compare the influence of patient location; home (H) or nursing home (NH). The results will allow improved control and management of CT-regimen; identifying non-compliers, optimising delivery, and potentially the control of possible non-compliance would therefore be optimised.

## Materials and methods

### Study design

Cohort prospective observational study, conducted from January 2018 to January 2019 following patients with CT treatment for OD who were included in the NDU data base for home enteral nutrition management.

### Study population

The sample was calculated based on an expected adherence of 50% [24, 25], with 95% confidence and a precision of ± 7%. Based on these assumptions, a minimum sample of 196 individuals was estimated. Considering a 20% loss rate, the required sample was 250 individuals. From the total number of patients in our database (667 at time of sample selection), 258 patients were selected, using a random number generator without repetition on-line.

We set the inclusion/exclusion criteria as follows:

#### Inclusion criteria

- Patients included in the NDU-database with specific treatment for OD with commercial thickener.
- Signature of informed consent (patient or legal guardian).

#### Exclusion criteria

- Patients who do not want more exhaustive control of the treatment but want the control carried out so far.
- Patients who reject the artificial food thickener.
- Non feasible telephone follow-up.
- Discharges; cessation of treatment for improvement, transfers or referrals.
- CT patients without established viscosity indication.

The study was approved by the Ethical Committee for Clinical Research of the Bellvitge University Hospital of Barcelona (December 2017) PR346/17 (CSI 17/51).

### Procedure

#### CT treatment

Patients with different pathologies who benefit from CT treatment for OD were identified in various settings hospital admissions, outpatient clinic, or in primary care. A validated volume/viscosity test [26], was performed to aid determine the safest and most effective volume and viscosity; nectar, honey or pudding. At the time of the study, the thickener available was starch-based (Nutilis®). Table 1 specifies the dosage recommended by the manufacturer, which will allow us to compare the consumption (number of cans) made by the patient with the expenditure that should be made according to the guideline.

**Table 1:** Dosage of commercial thickener used (self-made according to manufacturer indications)

Dosage of commercial thickener (Nutilis®; available in cans of 300 g). (Average liquid consumption of 5 glasses (200 ml)/day (1L)). [Range of 3–8 glasses/d.]		
Viscosity	Daily average (day) consumption	Length (days)/consumption of cans
**Nectar**: 1 scoop = 4 g = 2,dessert spoons (ds)	20 [12-32] g/d.	15 [10-25] d.
**Honey**: 1.5 scoop = 6 g = 2.5 ds.	30 [18–48] g/d.	10 [7-17] d.
**Pudding**: 2 scoops = 8 g = 4 ds.	40 [24–64] g/d.	8 [5-13] d.

Ordinarily nursing homes had to contact our NDU by mail to request CT delivery, indicating patient and dosage. At home this request was made by telephoning each patient quarterly. For this study, both locations were called separately.

### Control calls

Within the year, a NDU dietitian made 4 calls, one every 3 months: Call1 at start (C1), Call 2 (C2) at 3rd month, Call 3 (C3) at 6th month, and Call 4 (C4) at 9th month to every patient and/or carers, with a total follow up of 9 months. Data was collected in each call for study registration filling all parts of a questionnaire specially designed for this study. The calls were made by the same NDU dietitian with each lasting 10–20 min.

### Adherence study

Adherence is described by an international review for taxonomy as a conjunction of these terms: 1-Initiation of the guideline, 2- Implementation and 3- Persistence [20]. Our study is based on this terminology with adaptation to analysis of the adherence of CT in a population with OD:

1. *Initiation* of treatment which is considered from data registered in the NDU-database for each patient that is the data of initiation of CT.
2. *Implementation* defined as the extent to which the actual guideline reported by the patient matches the prescribed guideline. This has been assessed crossing the results of a questionnaire that measures the adequacy of CT use, related to the indication established by the health professional, with the evaluation of CT consumption (product delivered to patients from Pharmacy service). This is an indicator that approximates compliance with the indications made by the health professional.
3. *Persistence* defined as the time the patient is on treatment continuously [19]. This involves taking into account the term discontinuation of treatment which is the time the patient stops treatment [20]. In our study we have taken into account patient behaviour and we have counted the days they had been on treatment subtracting the estimated days without treatment (information referred by patients every three months).

### Questionnaire development

The questionnaire designed for this study was developed by dietitians from NDU, main managers of thickener dispensing (Table 2). It includes a variety of questions, ranging from dichotomous (yes/no) or quantitative types. These questions were intentionally repeated in different formats throughout the test to address inconsistencies that could arise. They were checked by the rest of the professionals who are part of the Hospital dysphagia committee; otorhinolaryngology, rehabilitation, geriatric, neurology and digestive physicians, nurses and speech language therapist, and also by staff from the pharmacy service, and externally by a professional expert in dysphagia from another hospital.

**Table 2:**
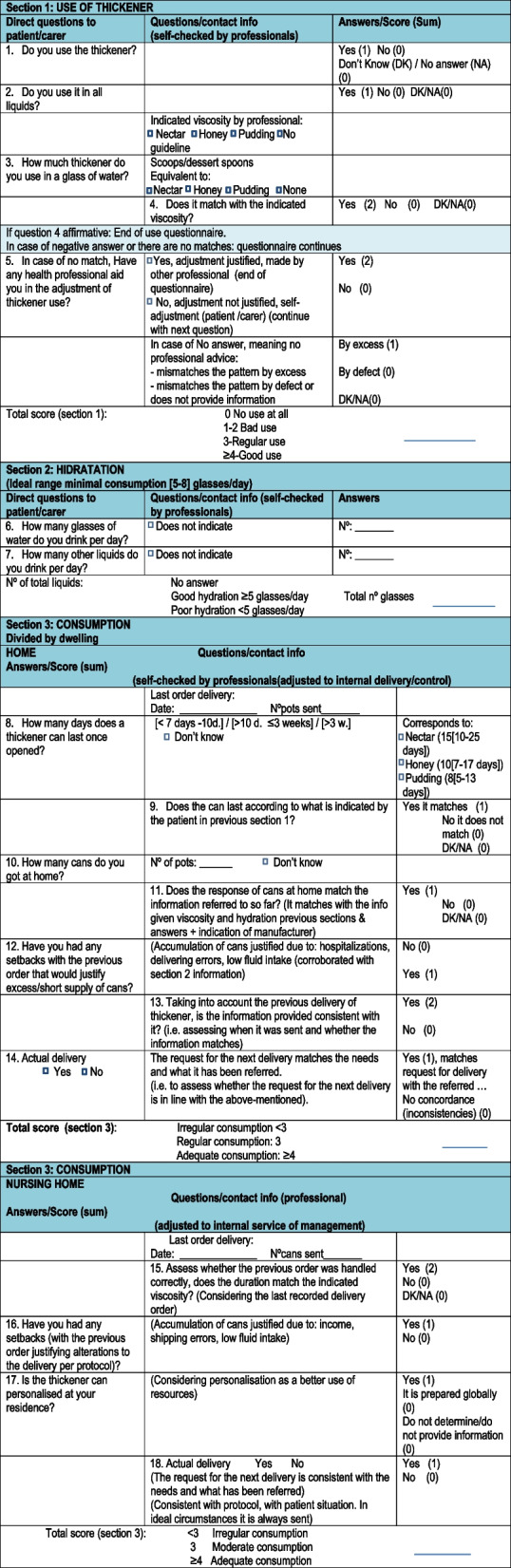
Questionnaire

The questionnaire is divided into three sections. Section 1 was designed to assess use of CT considering where the patient uses CT and follows the guidelines given by health professionals. This section comprises 5 questions, and the total score ranges from 0 to 4. The highest score indicates good use, meaning adherence to indicated guideline. A score of 3 indicates moderate use (use that needs improvement as answers failed in some of the questions such as no using CT in all liquids or fail in the correct dosage). The range 0–2 reflects poor use, failing in both use in all liquids and dosage (0 being no use at all).

The second section assesses hydration by considering water intake and total fluid intake, as these will influence the overall assessment of CT use and consumption hence global adherence. There are variations in the general recommendations for water intake. For the elderly they have been estimated at 1.0-1.5 L/day-3 L/day [27]. Considering these recommendations in 200 ml glasses would be equivalent to an intake of 5-7.5-15 glasses per day. We therefore consider an intake greater than or equal to 5 glasses/day to be correct. A low consumption of liquids is taken into account for the evaluation of the next section as it can justify a low consumption of CT.

The third section assesses CT consumption, which refers to the cans that patients order and receives (recorded by our service and checked by pharmacy). This is an indicator that may or may not support compliance with the indications made by the health professional. This section is very specific to each hospital as dispensing management of CT varies therefore these questions are based in our internal CT management process. We divided the questionnaire according to the place of residence as the management of the thickener differs. At H, it is possible to obtain more accurate information of certain aspects such as how long does the can last or how many cans are left. This is not possible at NH where CT cans may dispensed more disorderly, cans might be used individually in the NH, but often one can might be used for several patients. Therefore, special emphasis is placed on the fulfilment of the requests. The questionnaire comprises seven questions for home patients and four tailored for residential patients. Most of the answers are dichotomous where with each answer assigned a value of “1” or “2” (favourable response) or “0” (unfavourable response). We defined an inadequate consumption pattern when the sum of the responses was < 3, a moderate pattern = 3 and an adequate pattern when ≥ 4. Within the responses, possible setbacks that may have modified the consumption of CT; hospital admissions, dispatch errors, reduced fluid intake, were considered. Poor consumption was considered when the CT deliveries did not match with data described in Sect. 1. Moderate consumption was determined when there as an alignment but there were inconsistencies in some aspects and, finally a good consumption was when all aspects matched.

### Implementation

To analyse patient behaviour of guidelines implementation, we have combined the results of Sect. 1 (CT Use) and Sect. 3 (CT consumption) as shown in Table 3.

**Table 3:**
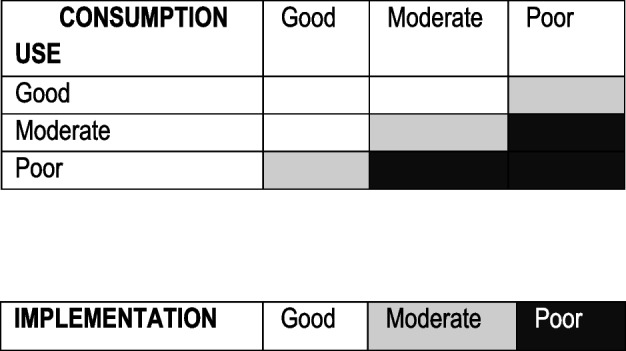
Algorithm of implementation

- Good implementation:
  - When CT use was good and consumption was good or moderate.
  - When CT use was moderate and consumption was good.
- Moderate implementation
  - When CT use was good, but consumption was poor.
  - When use was moderate and consumption was poor.
  - When use was poor but there was a proper consumption (not common).
- Poor implementation
  - When use was poor and consumption was moderate or poor.
  - When use was moderate and consumption was poor.

We have checked for improvement or worsening of use, consumption, and implementation between C1 and C4 according to the following criteria: Improvement, change from poor to moderate/good or moderate to good. Worsening was changing from good to moderate/poor or from moderate to poor.

### Validation questionnaire

The readability of the questionnaire was initially assessed using INFLESZ, readability software in Spanish [28]. Specifically, Sect. 1 (use) and Sect. 2 (number of liquids) and Sect. 3 (consumption) were subjected to a validation process. The content validity of these sections was determined using the Fehring Model [29]. This allows professional experts to assess whether each question is considered fit for purpose (representativeness for the CT adherence consultation) using a 5-point Likert scale; from strongly disagree (= 0) to strongly agree (= 1). The questions were scored with a simple sum or mean of the responses to the questionnaire ranked from 0 to 1 (0-0.25-0.5-0.75-1). The mean rating for each question, to be accepted as adequate, must be ≥ 0.8. A total of 15 professionals participated in this evaluation and the mean scores for the 6 questions ranged from 0.8 to 1 in all 3 sections. The information in Sect. 3 relates to the functioning and organisation of our centre and is therefore very specific. For this reason, it was only decided to reach a consensus on its content among the NDU professionals.

Sections 1 and 2 were tested for feasibility: A group of 30 patients were tested for internal consistency using Cronbach’s alpha; a score of 0.73 was obtained, showing an appropriate consistency. In the same group, a test-retest reliability analysis was performed by checking the responses of other dietitians in the team 15 days after they were first asked by another dietitian. The concordance analysis was measured with the Kappa test. The question “use of thickeners in all liquids” obtained a Kappa of 0.51 (moderate agreement) and the rest of the questions between 0.76 and 1 (good and very good agreement). The analysis of fluid intake between the answers of the first questionnaire and the next one was performed with Spearman’s correlation test. The “number of glasses per day” obtained an r = 0.7 and the “number of glasses of other liquids” an r = 0.8 (both with *p* < 0.0001).

### Variables

In C1, the following variables were included: Age, gender, diagnosis, highest educational level achieved (no formal education, primary, secondary or university education), person managing the thickener (patient, relatives or caregivers), dwelling/location (H or NH), days on CT (start of treatment in our centre), and indicated viscosity guideline at the time of inclusion in treatment (nectar, honey, pudding). An adherence questionnaire was completed on each call. Improvement or worsening of use, consumption and implementation was tested between C1 and C4 (change in patterns within groups). Finally, reasons for non-adherence; dislike of CT, perception of improving in deglutition (patient or carer) and no specific reasons were recorded.

### Statistics

We contrasted different dimensions established in our questionnaire to check adherence in our sample. We analysed patients with an indicated viscosity to check for a correct implementation. The comparison of qualitative variables has been carried out using the Chi-square test, and for the evolution of qualitative variables McNemar. Student’s t-test was used for the comparison of means. The Anova test was used to compare more than two means. Statistics have been calculated with Spss® programme (SPSS (IBM SPSS Statistics v 23).

## Results

### Descriptive data

Initially 258 patients were randomised from the NDU database. From these, 54 were excluded: 81.5% were deceased, 13% transferred to other areas, 4% ended treatment and 2.5% were untraceable. This resulted in 204 active patients with CT, of which 17.5% had no indicated guideline, leaving a final study sample of 82.4% (168). Main characteristics and alterations in the study population are displayed in Table 4. No differences found in the dwelling/location throughout the period. Mean age of 82.6 ± 11.1 years (H: 82.4 ± 9.9; NH: 82.8 ± 12.2; NS). Mostly women 57.8% with significant differences of proportion between H and NH (46.4% at H vs. 67% at NH, *p* < 0.01). Both age and gender distribution has remained similar throughout the study. Also, there were no significant differences with the population excluded from the study (with no indication of viscosity): Average age 83.3 ± 13.2 years and 61% women. Mean days on thickener before the study were globally of 509 ± 475.28 [0-2762] days; at home 479.57 ± 473.45 days and in residence 531.78 ± 477.51 with no significant differences found between them.

**Table 4:**
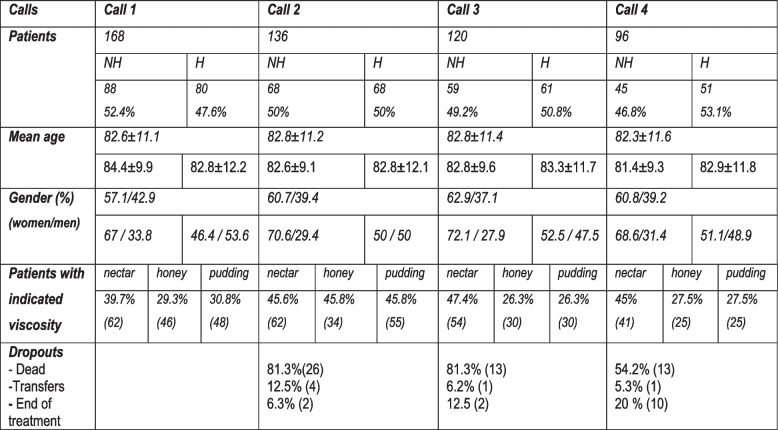
Descriptive data in successive calls

The main diagnostics were neurology based (stroke, dementias mainly) 91.7%, respiratory 3.4%, oncology 2.5%, digestive 1% and other pathologies 1.5%. Approximately half of the attended patients, 50.5%, came from the hospital; 47.1% from primary care and 2.5% from other hospitals. Educational level in the person managing the CT was not reflected in 6.9% of the sample. Significant difference was found in distribution of education level according to dwelling (*p* < 0.001): At home 16% had no formal studies vs. 1% at NH, 37% primary studies vs. 3% at NH, with secondary education 25% at H vs. 11% at NH and 18% had university studies at H vs. 77% of nursing staff at NH. There was also significant difference among managers of the thickener, but this was mainly due to the fact that at NH 97.4% were carers (2.6% relatives). At H it was the patient in 14.6%, relatives 64% and carers 21.4%; (*p* < 0.001).

### Adherence

During the 4 calls data had been obtained in each section of the questionnaire (Table 5)*.* In C1 42.3% of good use was found, while 26.8% reported poor use. However, in C4, values displayed a different distribution primarily due to a shift in the moderate group, resulting in increased values for poor use from 26.8 to 52.1%, *p* < 0.01 (C1 vs. C4). Relating to consumption there was a difference data distribution with better values for good consumption in C4, and especially lower values for poor consumption in the last call which decreased from 36.9 to 28.1%, *p* < 0.01. Relating to implementation, we observed overall values of 50% good implementation in C1 and 55.2% in C4, and increased values for poor implementation from C1 to C4 29.8% and 38.5% respectively, and with a lower percentage in the moderate group, *p* < 0.01. A detailed breakdown of the implementation data in C1 can be seen at Table S1. (All implementation data have therefore been obtained by combining the use and consumption values).

**Table 5:** General values in successive calls in patients with guidelines given

		**CALL 1** **(168)**	**CALL 2** **(136)**	**CALL 3** **(120)**	**CALL 4** **(96)**
**Use**					*
	*Good*	42.3% (71)	44.9% (61)	49.2% (59)	41.7% (40)
	*Moderate*	31% (52)	20.6% (28)	17.5% (42)	6.3% (6)
	*Poor*	26.8% (45)	34.6% (47)	33.3% (32)	52.1% (50)
**Hydratation**^**a**^	*Good*	71.48% (90)	65.5% (76)	72.1% (98)	71.6% (68)
	*Poor*	28.6% (36)	34.5% (40)	27.9% (38)	28.4% (27)
**Consumption**					*
	*Good*	46.4% (62)	56.6% (77)	45.8% (55)	55.2% (53)
	*Moderate*	16.7% (28)	12.5% (17)	21.7% (26)	16.7% (16)
	*Poor*	36.9% (78)	30.9% (42)	32.5% (39)	28.1% (27)
**Implementation**					*
	*Good*	50% (84)	40.4% (55)	48.3% (58)	55.2% (53)
	*Moderate*	20.2% (34)	27.2% (37)	14.2% (17)	6.3% (6)
	*Poor*	29.8% (50)	32.4% (44)	37.5% (45)	38.5% (37)

*Comparison with Call 1: *p* < 0.01

^a^Data obtained in 75% of sample

Analysing distribution according to dwelling in C1, values showed a significant better use and consumption patterns of NH compared to H, (*p* < 0.05) (data shown in Table 6). These values are different in C4 where values were better for H. Relating to implementation; distribution pattern in C1 resulted non-significant although a better percentage of good implementation was observed in NH compared to H. However, in C4, there were significant differences in implementation between NH and H with better values for H subjects.

**Table 6:** General values in successive calls in patients with guidelines given according to dwelling

		**CALL 1** **(168)**		**CALL 2** **(136)**		**CALL 3** **(120)**		**CALL 4** **(96)**	
		**H** **(80)**	**NH** **(88)**	**H** **(68)**	**NH** **(68)**	**H** **(59)**	**NH** **(61)**	**H** **(45)**	**NH** **(51)**
**USE**	**G**	43.8% (35)	40.9% (36)	51.5% (35)	26.5% (18)	52.5% (31)	45.9% (28)	66.7% (30)	39.2% (20)
	**M**	18.8% (15)	42% (37)	5.9% (4)	35.3% (24)	22% (13)	13.1% (8)	4.4% (2)	7.8% (4)
	**P**	37.5% (30)	17% (15)	42.6% (29)	38.2% (26)	25.4% (15)	41% (25)	28.9% (13)	52.9% (27)
		*p* < 0.05		*p* < 0.001		*p* < 0.001		*p* < 0.02	
**HYD**^**a**^	**G**	65.6% (42)	37.4% (48)	70.4% (28)	61.3% (78)	80.9% (38)	70.93% (39)	72.7% (32)	70.6% (36)
	**B**	34.4% (22)	22.6% (14)	29.6% (16)	38.7% (24)	19.1% (9)	29.1% (16)	27.3% (12)	29.4% (15)
		NS		NS		NS		NS	
**CON**	**G**	37.5% (30)	54.5% (48)	25% (17)	48.5% (33)	44.1% (26)	47.4% (29)	66.7% (30)	47.1% (24)
	**M**	16.3% (13)	17% (15)	20–6% (14)	22.1% (15)	16.9% (10)	26.2% (16)	6.7% (3)	23.5% (12)
	**P**	46.3% (37)	28.4% (25)	54.4% (37)	29.4% (20)	39% (23)	26.2% (16)	26.7% (12)	29.4% (15)
		*p* < 0.05		*p* < 0.01		NS		*p* < 0.05	
**IMP**	**G**	37.5% (30)	58% (51)	36.8% (25)	26.5% (18)	47.5% (28)	49.2% (30)	57.8% (32)	56.8% (21)
	**M**	21.3% (17)	19.3% (17)	19.1% (13)	27.9% (19)	18.6% (11)	9.8% (6)	0% (0)	6% (6)
	**P**	41.3% (33)	22.7% (20)	44.1% (30)	45.6% (31)	33.9% (20)	41% (25)	24.4% (13)	17.6% (24)
		NS		NS		NS		*p* < 0.01	

*HYD* hydration, *CON* consumption, *IMP* implementation, *G* good, *M* moderate, *P* poor

^a^Data obtained in 75% of sample

A total of 96 patients remained during the whole following process (at C4). We analysed alterations in distribution patterns from C1 to C4 (results can be seen at Table S2). The primary finding was that patients at H showed no significant changes in the distribution patterns of use, consumption and implementation. However, a significant deterioration in CT use and implementation was observed at NH patients: Specifically from the ‘good use’ group and ‘good implementation’ group in C1, only 47.8% and 40.6%, respectively remained within this range in C4, the rest exhibited worsening patterns.

Regarding behavioural changes (group upgrading) (results in Table 7), an improvement of CT use was found at H (24.4%) versus NH (17.5%), (*p* < 0.05). There were no significant changes in consumption although the improvement value at H was higher at 42.2% compared to NH, which had a value of 29.4%. Finally, a significant improvement in implementation was observed among patients living at H, with a value of 31.1%, while those living at NH presented a value of 15.7% (*p* < 0.05).

**Table 7:** Changes occurred according to dwelling from C1 to C4

		**No changes**	**Improvement**	**Worsening**
Use	H (45)	57.8% (26)	24.4% (11)	17.8% (8)
	NH (51)	37.2% (19)	17.6% (9)	45.1% (23)
		Test: *p* < 0.05		
Consumption	H (45)	42.2% (19)	42.2% (19)	15.5% (7)
	NH (51)	37.2% (19)	29.4% (15)	33.3% (17)
		Test: NS		
Implementation	H (45)	55.5% (25)	31.1% (14)	13.3% (6)
	NH (51)	39.2% (20)	15.7% (8)	45.1% (23)
		Test: *p* < 0.05		

No significant differences in usage were observed according to the number of days patients were on CT prior to the study. In the group with good use, average days with CT were 549.11 ± 485.78d, in the moderate use group of 448.50 ± 409.27d and in the bad use group 440.76 ± 465.43d. There were also no differences in the mean number of days with previous CT according to the degree of consumption or implementation. When categorizing the number of days with CT into tertiles, consumption assessment was significantly better in the third tertile (Table S3). There were no significant differences observed in use and implementation among the different tertiles. However, there was a trend toward better use in the upper tertile compared to the lower one (50% vs. 35.7%).

When examining the relationship between the recommended viscosity and adherence, in C1, it was observed that the use of CT was good in 26.3%, 59.5%, and 52% of cases with nectar, honey, and pudding viscosities, respectively (*p* = 0.001). Likewise, the consumption of CT was good in 44.7%, 38.1%, and 56% of cases with nectar, honey, and pudding viscosities, respectively (*p* = NS). Although without statistical significance, implementation was good in 40.8%, 57.1%, and 58% of cases with nectar, honey, and pudding textures, respectively. Further details regarding the relationship between viscosity type and adherence can be found in supplementary Table S4. The main finding is that viscosity did not appear to influence implementation or consumption, but there was a significantly better usage of CT in cases with honey viscosity (59.5%) and pudding 52% versus cases with nectar (26.3%).

Finally, educational level was studied only at H and we did not find any significant differences in its influences in any of the sections (*data not shown*).

### Liquid consumption

Liquid consumption data was obtained in 75% (126) of the studied sample (168). In C1 there was a mean consumption of total liquids of 5.6 ± 1.9 [1–8] glasses (gl) per day, with no differences in following calls (C2: 6.36 ± 2.73; C3: 6.29 ± 2.63; C4: 5.42 ± 2.23). Analysing data in C1 and separating by type of dwelling there were no significant differences between them: At H consumption of total liquids was 5.42 ± 1.87 gl/d and 5.81 ± 2.03 gl/d at NH. Also there were no differences throughout the period studied (C2: 6.43 ± 2.59 H / 6.18 ± 2.66 NH; C3: 6.43 ± 2.59 H / 6.18 ± 2.67 NH; C4: 5.39 ± 2.39 H / 5.45 ± 2.10 NH).

Considering water consumption there was 61% (94) who did not consume enough water, but this is compensated for by the consumption of total liquids resulting in overall good liquid consumption. There are no remarkable changes in this consumption throughout calls (Table 5). Separating by place of residence, 68% (49) had good hydration at H and 69.5% at NH, no differences observed.

### Persistence

Patients from our sample maintained an average of 771.87 ± 501.52 [42-3097] days with CT. This sample showed a very good persistence of 89.7%, only 10.3% (21) of patients showed discontinuation process. From this 3.8% (8) kept discontinuation in the whole period.

### Reasons for lack of adherence

There are 108 patients who give reasons of lack of adherence. The first one is dislike with CT in 59.4% (*n* = 63), secondly no specific reasons, including lack of perception of a problem in swallowing in 27.4% (*n* = 29) and patients or carers who feel objective improvement (with no professional counselling) 13.2% (*n* = 14).

## Discussion

This study examines adherence to CT treatment in patients with OD. Initially at call 1, a 50% adherence rate was found and 55% at last call during the whole study period. Patients who exhibited good adherence from the beginning remained stable, while the most significant changes occurred within the moderate compliance group. These changes were more favorable for home-dwelling subjects than for those living in nursing homes. The observed sample consisted of a geriatric population with neurological main diagnoses such as dementia and stroke. The majority were women, particularly in nursing homes. The data analysis from the studied sample support that OD is a chronic condition, with an average CT use in the sample of 1.4 years. Notably, some patients had been using it for as long as 7.5 years.

A proper viscosity guideline was indicated in the majority of the sample, although there are still patients who take CT whom an appropriate viscosity has not been indicated (17.5%). This lack of a proper diagnosis which leads to inadequate treatment, is a frequently reported issue, and it has been described in the literature [10]. In our sample, nectar was the most indicated viscosity which is good as clinicians are encouraged to prescribe the minimal level of thickness needed for swallowing safety [28]. This is to optimize treatment and also because a precise diagnosis with the minimum CT is best, as the intake of thickened liquids seems to be perceived as unpalatable, and patients prescribed overly thick liquids may show a lower compliance [16, 29]. In our study this is not the case, as we found a significantly better use in those with honey, followed by pudding viscosities probably due to an increased perception of swallowing problems and the need of CT.

Globally, based on our treatment implementation criteria (relating CT use to consumption), we achieved a 50% adherence rate on the first call, which arises to 55% in last call. This aligns with a review of studies on adherence to OD recommendations, covering various approaches like diet, postures, and fluid viscosities, with rates spanning 21.9–84% [10]. However, it is important to note that these studies exhibit significant heterogeneity due to variations in settings, pathologies, different treatments for OD and differences in the collection of data. Within adherence reviews there are many articles in head and neck cancer which has a different therapeutic component from neurological pathology. In this sense, it was not compared to these rates because cancer population features are unique and cannot be easily generalised to other dysphagia-related conditions [10]. Hospital studies examining fluid adaptation compliance have revealed adherence rates in acute wards of 48%, which improve to 64% when staff underwent specific training [24]. Another study revealed 88% compliance at OD sensitized wards [30].

In outpatient settings, focusing on CT treatment, which is our main objective, we compare our results with various studies. Firstly, a Korean study [25], reported an overall adherence rate (in terms of use) for liquid texture modification of 56.5%. Specifically, their outpatients adherence rate was 40.5%, aligning with our results of 42% found in Sect. 1 (considering only adherence in terms of use). However, it is important to note that this study is retrospective, conducted with a small sample and lacks specific guidelines on viscosities. In this context, we best compare our results with a published Spanish thesis [30] which takes into account specific guidelines on viscosities similar to ours and follows patients at discharge from the hospital, showing an adherence rate of 55.5% over an 18-month follow-up period. Moreover, a recently published Spanish work has revealed an increase in adherence rates at discharge, with a rate of 76% adherence to fluid adaptation after a month of discharge [31]. However, authors described subjectivity, as it relies solely in just one call (self-report). Also, Low et al. [17] studied 86 OD outpatients, mostly with neurological pathology, finding a good compliance of indications in the CT use in 84%. The type of questionnaire done and the fact that is retrospective could explain the difference with our study.

The place of residence could also influence adherence. Indeed, in the present study remarkable differences were observed between H and NH. At baseline, worse adherence was observed in H patients compared to NH patients. However, over the course of the study, a significant improvement was observed in H cases and, conversely, a worsening in NH cases. There are few studies comparing adherence to CT according to place of residence and the results are not homogeneous. Low et al. [17] observed similar results (59% in NH vs. 41% in H) to ours in terms of implementation (58% in NH vs. 37.5%), although they do not provide follow-up data. Another recent study also observed better adherence to CT in NH at one month after hospital discharge [31]. However, Espinosa [30], while detecting high adherence to the recommendations during hospital admission (about 88%), at discharge follow-up it drops by almost half and, in contrast to the present study, no significant differences were observed between H and NH. Probably, the difference with our study is due to the fact that, in each of our four calls, a comprehensive questionnaire was carried out which could favour a positive change, especially in the cases of H. In NH, they were more reluctant to changes, perhaps due to the fact that personnel are more subject to changes and work overload than caregivers at home. Our work support that with intervention this can be reversed as more positive changes were made in patients at home, showing a greater flexibility and higher potential for adaptations compared to those at NH who were more reluctant to changes. This matter was established in a previously published paper in relation to our findings on diet textures and viscosities which demonstrated more rigid patterns at NH [32].

Another factor influencing compliance may be time. On the one hand, we demonstrated that the duration of the patients’ CT treatment did not interfere with its implementation. In fact, there was an improvement in implementation (especially in consumption) in the third tertile. This implies, in our study, than those with CT for longer showed better results. On the other hand, we observed changes throughout the study period, primarily among patients who did not adhere to the treatment properly from the beginning. These changes were more favorable for patients at H, as we mentioned earlier. This is aligned with Espinosa [30] who also describes this group that needs reinforcement and emphasizes the importance of establishing a good treatment plan from the outset. This underscores the need to provide more information and ensure patients understand the implications of non-compliance to the treatment.

In relation to reasons for adherence, it was generally confirmed that the primary reason for non-adherence was a dislike of CT (59.4%), aligning with findings reported elsewhere. In this sense, it has been described that starch thickeners such as the one used in our study have been found to leave a grainy texture and a certain starchy taste especially in nectar and honey [28]. Probably the new versions of xanthan gum-based thickeners will have a positive influence although they have also reported to be rougher than the previous ones [28]. Both types of thickening agents have their advantages and disadvantages; nevertheless, the treatment our patients are familiar with is the starch-based, as we have not introduced the newer version just yet. Among other aspects described of lack of adherence we found the lack of perception to be a problem (27.4%). This is described specially in stroke patients who rarely perceive that they have a swallowing problem [33]. It should be noted that there is a subgroup of patients (17.5%), who do not have an indicated regimen, a situation commonly described for OD [10]. It was considered that there was a great variability in this situation and therefore it could not be determined whether they follow specific guidelines. Yet we believe it is crucial for healthcare professionals to supervise and describe the situation of OD patients to ensure appropriate care, with a proper diagnosis and management with adequate treatment and following. There is a great need to raise awareness about the identification and treatment of OD and a better adequacy of defined treatments [34]. Also, it would be considered to be essential to focus on initial better information and a proper follow up to aid in overall adherence to treatment been.

Our results show a good persistence, and it has not been found an influence in the time being with the CT treatment. This has to be taken into consideration as CT adherence may differ which is supported by evidence of studies that show that long-term treatment deteriorates even when initial adherence is good [17]. In fact we have studied the bias time may entail in two ways: On the one hand, we compared the means of he days with CT in the three adherence groups, with no difference, and on the other hand, we grouped the sample by tertiles of days with CT, and a trend towards better adherence was even observed in the upper tertile.

In terms of water assessment it is difficult to assess the actual total fluid intake and relate it to hydration as many factors are involved in the requirements such as muscle mass, physiological circumstances etc. [33]. There is currently no consensus on the most correct methodology for assessing total fluid intake [34]. A 24-hour count may not be representative as there are many variations in requirements but there is no validated method and within nutrient analysis it is a widely used resource [35, 36]. In our study we identified the estimated amount of water and fluids per patient and found that water intake is low but when total fluids are added up, a good minimum intake is achieved. Also we did not find interference with CT in liquid consumption but we definitively need to investigate this issue more thoroughly.

In our work we encountered some limitations that should be considered. First of all, there is a limitation of relying on self-reported information from patient or caregivers which can introduce bias. Additionally, it is difficult to obtain information on the daily use of CT, which can limit the strength of any findings in adherence to this kind of treatment. Telephone call problem is accentuated in nursing homes, the interlocutors during calls may differ, and despite including questions in the questionnaire to monitor for inconsistencies, this is likely to have introduced some bias in the results. Also there is a limitation in comparing works as there are differences in approaching OD, we have tried to compare it to similar studies and we think sharing our work can give information and highlight that our Sect. 1 (‘use section’) has undergone validation and can serve as a reliable source for future comparisons in CT adherence. Related to this remark a positive aspect of the study is the development of a structured and validated questionnaire which has been developed especially to relate actual intake of CT with real expenditure from Pharmacy records which allows to a better control.

Finally a positive aspect of the study is that it is a prospective study with a considerable homogenous sample size, which provides information on the behaviour of patients undergoing CT treatment and also it describes differences in the place of living. As a result, we believe that calls have a positive impact on improving adherence. It is possible that the longer duration of these calls (with a structured questionnaire) may have contributed to this improvement. However, it is worth noting that similar calls were conducted for NH, suggesting that we may need a different approach for these locations. Also, the trend to obtain better values for upper tertiles in consumption patterns at the beginning assures us that telephone feedback is beneficial. Finally it helps us to realize that closely assistance effectively enhances adherence. Obviously there is a need for more studies to investigate ways of improving patient care in OD, especially at NH where persons can remain for many years and their treatment must be revised.

## Conclusion

The study concludes that adherence to CT indications is generally low in the examined area. It underscores the importance of closely monitoring adherence, focusing on interrelating use and consumption, which could serve as a valuable parameter to compare adherence to CT in OD patients in future studies (through a validated questionnaire). There were many shifts throughout the study period with patients who initially followed guidelines remaining stable. In contrast, those with poorer performance, especially in the moderate group, showed positive changes at home but deterioration in nursing homes. The study highlights the importance of providing quality information at hospital discharge and a proper follow-up, as poor discharge communication could lead to inappropriate post-hospital dysphagia care, with resultant aspiration pneumonia and need for costly re-hospitalisation [37]. Additionally, it suggests that telephone calls enhance adherence but may not be sufficient for more specific changes, especially in NH where there appears to be a greater need for developing strategies for OD monitoring. In summary, consistent reinforcement, investigating causes of non-adherence, and adjusting to the patient’s situation are emphasized for long-term management improvement in dysphagia patients.

### Supplementary Information

**Additional file 1:** **Table S1.** Implementation in call 1. **Table S2.** Compared distributions between C1-C4 by dwelling. **Table S3.** Tertiles of time with CT at the begining of the study. **Table S4.** Adherence according to the indicated viscosity guideline.

## Data Availability

Data is available from the individual publications included in the reference list. Further detail is available from the corresponding author on reasonable request.
