# Supplementary material for: Adherence to commercial food thickener in patients with oropharyngeal dysphagia
Source: BMC Geriatr. 2024 Jan 16;24:67. doi: 10.1186/s12877-023-04589-4 (PMC10792797; doi:10.1186/s12877-023-04589-4)
Supplement: Supplementary file 1 — Additional file 1: Table S1. Implementation in call 1. Table S2. Compared distributions between C1-C4 by dwelling. Table S3. Tertiles of time with CT at the begining of the study. Table S4. Adherence according to the indicated viscosity guideline. [file 12877_2023_4589_MOESM1_ESM.docx]

**Supplementary tables**

***Table 1S.*** Implementation in call 1

| CONSUMPTION 1 | | Good | Moderate | Poor |
| --- | --- | --- | --- | --- |
| USE1 | Good | 57.7% (41) | 18.3% (13) | 30% 17 |
|  | Moderate | 57.7% (30) | 19.2% (10) | 23.1% (12) |
|  | Poor | 15.5% (7) | 11.1% (5) | 73.3% (33) |

| **IMPLEMENTATION** | Good  50% (84) | Moderate  20.2% (34) | Poor  29.8% (50) |
| --- | --- | --- | --- |

***Table 2S***. Compared distributions between C1-C4 by dwelling.

|  | | C4 | | |
| --- | --- | --- | --- | --- |
| **USE**  **HOME** | C1 | Good  (30) | Moderate  (2) | Poor  (13) |
|  | G (25) | 80% (20) | 4% (1) | 16% (4) |
|  | M (7) | 57% (4) | 0 | 43% (3) |
|  | P (13) | 46% (6) | 7.8% (1) | 46.2% (6) |
|  | | Mc Nemar; p =0.362 | | |
| **USE**  **NURSING HOME** |  | G  (20) | M  (4) | P  (27) |
|  | G (23) | 47.8% (11) | 8.7% (2) | 43.4% (10) |
|  | M (21) | 38% (8) | 8.3% (2) | 52.4% (11) |
|  | P (7) | 14.3% (1) | (0) | 85.7% (6) |
|  | | Mc Nemar; p =0.000 | | |
| **CONSUMPTION**  **HOME** |  | G  (30) | M  (3) | P  (12) |
|  | G (20) | 65% (13) | 5% (1) | 30% (6) |
|  | M (5) | 100% (5) | (0) | (0) |
|  | P (20) | 60% (12) | 10% (2) | 30% (6) |
|  | | Mc Nemar; p=0.083 | | |
| **CONSUMPTION**  **NURSING HOME** |  | G  (24) | M  (12) | P  (15) |
|  | G (28) | 42.8% (12) | 25% (7) | 32.1% (9) |
|  | M(8) | 55.5% (5) | 25% (2) | 12.5% (1) |
|  | P (15) | 46.7% (7) | 20% (3) | 33.3% (5) |
|  | | Mc Nemar; p=0.663 | | |
| **IMPLEMENTATION**  **HOME** |  | G  (32) | M  (0) | P  (13) |
|  | G (24) | 75% (18) | (0) | 25% (6) |
|  | M (10) | 100%(10) | (0) | (0) |
|  | P (11) | 36.4% (4) | (0) | 63.6% (7) |
|  | | Mc Nemar; p=0.5 | | |
| **IMPLEMENTATION**  **NURSING HOME** |  | G  (24) | M  (6) | P  (24) |
|  | G (32) | 40.6% (13) | 12.5% (4) | 46.9% (15) |
|  | M (11) | 45.5% (5) | 18.2% (2) | 36.4% (4) |
|  | P (8) | 37.5% (3) | (0) | 62.5% (5) |
|  | | Mc Nemar; p=0.007 | | |

***Table 3S***. Tertiles of time with CT at the begining of the study

|  |  | **Tertile 1 (0-218 days)** | **Tertile 2 (219-590 days)** | **Tertile 3 (591-2762 days)** | ***p*** |
| --- | --- | --- | --- | --- | --- |
| **Use** | Good | 35.7% (20) | 41.1% (23) | 50% (28) | 0.630 |
|  | Moderate | 33.9% (19) | 30.4% (17) | 28.6% (16) |  |
|  | Poor | 30.4% (17) | 28.6% (16) | 21.4% (12) |  |
| **Consumption** | Good | 44.6% (25) | 41.1% (23) | 53.6% (30) | P=0.040 |
|  | Moderate | 14.3% (8) | 28.6% (16) | 7.1% (9) |  |
|  | Poor | 41.1% (23) | 30.4% (17) | 39.3% (22) |  |
| **Implementation** | Good | 48.2% (27) | 51.8% (29) | 50% (28) | P=0.234 |
|  | Moderate | 14.3% (8) | 17.9% (10) | 28.6% (16) |  |
|  | Poor | 37.5% (21) | 30.4% (17) | 21.4% (12) |  |

**Table 4S**. - Adherence according to the indicated viscosity guideline

|  |  | **Nectar (76)** | **Honey (42)** | **Pudding (50)** | ***p*** |
| --- | --- | --- | --- | --- | --- |
| **Use** | Good | 26.3% (20) | 59.5% (25) | 52% (26) | 0.001 |
|  | Moderate | 34.2% (26) | 23.8% (10) | 32% (16) |  |
|  | Poor | 39.5 (30) | 16.7% (7) | 16% (8) |  |
| **Consume** | Good | 44.7% (34) | 38.1% (16) | 56% (28) | NS |
|  | Moderate | 15.8% (12) | 23.8% (10) | 12% (6) |  |
|  | Poor | 39.5% (30) | 38.1% (16) | 32% (16) |  |
| **Implementation** | Good | 40.8% (31) | 57.1% (24) | 58% (29) | NS |
|  | Moderate | 19.7% (15) | 19% (8) | 22% (11) |  |
|  | Poor | 39.5% (30) | 23.8% (10) | 20% (10) |  |
